# Overwinter survival of *Corbicula fluminea* in a central Minnesota lake

**DOI:** 10.1371/journal.pone.0271402

**Published:** 2022-07-15

**Authors:** Megan M. Weber, Daniel Cibulka

**Affiliations:** 1 University of Minnesota Extension, Andover, Minnesota, United States of America; 2 Minnesota Aquatic Invasive Species Research Center, University of Minnesota, St. Paul, Minnesota, United States of America; 3 Sherburne Soil and Water Conservation District, Elk River, Minnesota, United States of America; University of California, UNITED STATES

## Abstract

Although *Corbicula fluminea* has been one of the more prolific freshwater invasive species in the world, previous studies have suggested a low probability for overwinter survival in northern latitudes without an artificially created thermal refuge. The discovery of live *C*. *fluminea* in a central Minnesota lake absent any known thermal refuge in 2020 presented an opportunity to further evaluate the overwinter survival and population structure of *C*. *fluminea* at the presumed edge of their potential range. The population was monitored from December 2020 through September 2021 alongside water temperature to better understand at which temperatures *C*. *fluminea* survived and if the population structure suggested reproduction occurring in the lake. We documented live *C*. *fluminea* in temperatures as low as 0.3°C. Shell size of recovered individuals suggested multiple cohorts, and the appearance of a new cohort at the end of the study, indicating active reproduction in the lake and suggesting the population had likely been present in the lake for at least two winters by the conclusion of the study period. Our findings provide evidence of the survival below historically documented lower lethal temperature limits and suggests adaptations to modeling predicting suitable habitat, both present and in a changing climate, are necessary to better assess risk of invasion by this species.

## Introduction

Invasive species (defined as introduced organisms that produce negative impacts outside their native range [1, 2] or introduced organisms that continue to disperse and reproduce across multiple sites beyond their native range [3]) have become increasingly prevalent with the rise of globalization through both intentional and unintentional means [4, 5]. The spread of invasive species has been a major driver of biodiversity losses across the globe [6, 7] and continue to put increasing pressures on biodiversity [8]. Additionally their introductions can result in threats to agricultural resources [9], alterations to hydrology [10], and economic impacts [11–13]. Haubrock et al. [14] note that invasive freshwater bivalves, in particular, have had substantial economic impacts globally with an estimated cost of $63.7 billion USD from 1980–2020.

*Corbicula fluminea* is a freshwater clam from the Family Cyrenidae and has a native range predominately located in Southeast Asia [15]. *C*. *fluminea* is capable of self-fertilization and is able to reproduce through a rare form of asexual reproduction called androgenesis, where only the paternal genetic material is included in development after fertilization [16, 17]. It is often cited as one of the most invasive freshwater species in the world [15, 18, 19] and is now found broadly across North America, South America, and Europe with limited, more recent records in Northwest Africa [20]. Vectors contributing to the spread of *C*. *fluminea* include human-mediated pathways such as release of unwanted aquarium pets, transport in ballast water, introduction via food systems, bait pathways, sediment transport, and recreational boating [15, 20–22]. Further dispersal after introduction is also mediated through natural downstream transport and potentially by fish (via survival through the digestive track) and waterfowl and shorebirds (through juvenile attachment or entanglement) [15, 21].

Climate modeling suggests *C*. *fluminea* is likely to experience an even broader range expansion globally in a warming climate [23, 24]. The first discovery in North America was in the 1920s and it has since spread along the Pacific coastal states, across the southern United States, and into much of the eastern United States with the exception of more northern latitudes [15, 25]. Impacts of *C*. *fluminea* in its invaded range includes infrastructure biofouling for raw water users [26] and associated economic costs for management [14], increased removal of seston from water bodies [27], zooplankton community shifts [28], and increased mortality of native mussel glochidia [29].

Mattice and Dye [30] established a lower temperature threshold of 2 °C for *C*. *fluminea* survival through laboratory experiments. This threshold has been widely cited and accepted in our literature review, though it is notably based on one experimental project using only clams collected from one location in Tennessee, USA [30]. Survival in northern climates has been limited to areas of thermal refuge such as effluent from power plant cooling towers or other industrial discharge to water bodies [15, 31, 32]. However, growing body of literature has suggested that *C*. *fluminea* may be able to survive in water temperatures below the 2 °C threshold. Some of these field-based studies lack direct water temperature data, but are in areas where waters are presumed to reach temperatures below 2 °C during winter months [33–36]. Massive die-offs have also been documented following extreme cold [31, 37], though some studies tracking these die-off events documented a subset of the population that was able to persist after exposure to temperatures below 2 °C [38, 39]. In the lab, more recent studies using temperature manipulation have suggested the temperature threshold for *C*. *fluminea* is actually lower than 2 °C [40] and that acclimation to lower temperatures improves the cold tolerance of *C*. *fluminea* [41].

Citizen scientists have played an important role in making important invasive species discoveries that lead to new knowledge about invasive species range expansion and distribution patterns [42–44]. Adding to this list of volunteer discoveries was a report of *C*. *fluminea* in August 2020 by a young volunteer participating in Starry Trek (www.starrytrek.org), an aquatic invasive species early detection event, in Briggs Lake, Sherburne County, Minnesota (USA). This marked the first reported occurrence of live *C*. *fluminea* in an inland lake in Minnesota. In this study, we documented the overwinter survival of *Corbicula fluminea* in Briggs Lake and characterized the population structure. This information helps provide in-situ data to support a growing body of evidence that the lower lethal temperature limit for *Corbicula fluminea* extends below 2 °C.

## Methods

**Fig 1:**
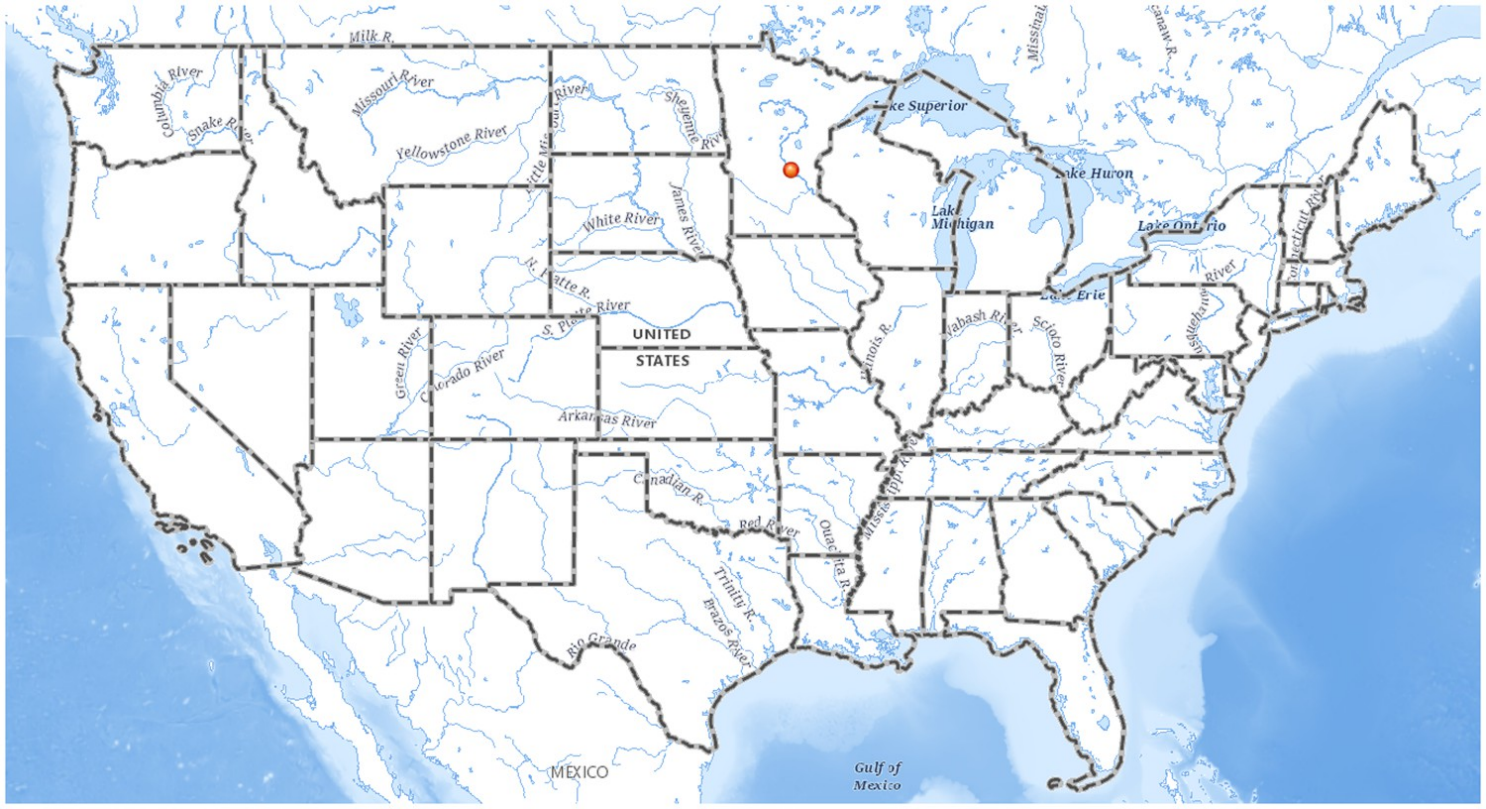
Study site map. The location of Briggs Lake is marked by a red dot. Map generated using USGS The National Map: National Hydrography Dataset. Data refreshed January, 2022 | USGS The National Map: National Boundaries Dataset. Data refreshed April, 2022.

This study was conducted at Briggs Lake, Sherburne County, Minnesota, USA (45.490449°N, 93.950095°W, Fig 1). Briggs Lake is a 404 acre eutrophic lake and the largest of the four-lake Briggs Lake Chain of Lakes found in central Minnesota, north-northwest of the town of Clear Lake. It is has a broad littoral zone with 54% of the lake being classified as littoral [45]. The study area included the space within 70 meters of the public boat launch, which is predominantly characterized by shallow water (generally less than 1.5 meters) and sandy substrate. The public boat launch was the area the initial discovery was made during Starry Trek and the area was selected after a scoping trip to Briggs Lake in October 2020. The scoping trip indicated that the *C*. *fluminea* population was limited to this part of the lake. Outside of the study area the substrates quickly transitioned from sand to being dominated by decaying organic matter.

We began monthly sampling at the study area in December 2020 as soon as sufficient ice cover to safely walk on the ice for sampling activities was present. Through-ice sampling continued through March 2021 and open water sampling was then conducted April 2021 through September 2021. We sampled ten haphazardly selected sites within the study area each month, with the exception of February 2021 in which we were limited to seven sites due to mechanical issues with the ice auger preventing further sampling that day. Sampling activities were covered under a Minnesota Department of Natural Resources Permit for Prohibited Invasive Species, Aquatic Plants, and Water Transport No. 575 and studies at the University of Minnesota involving invertebrates are exempted from review by the Institutional Animal Care and Use Committee (IACUC).

**Fig 2:**
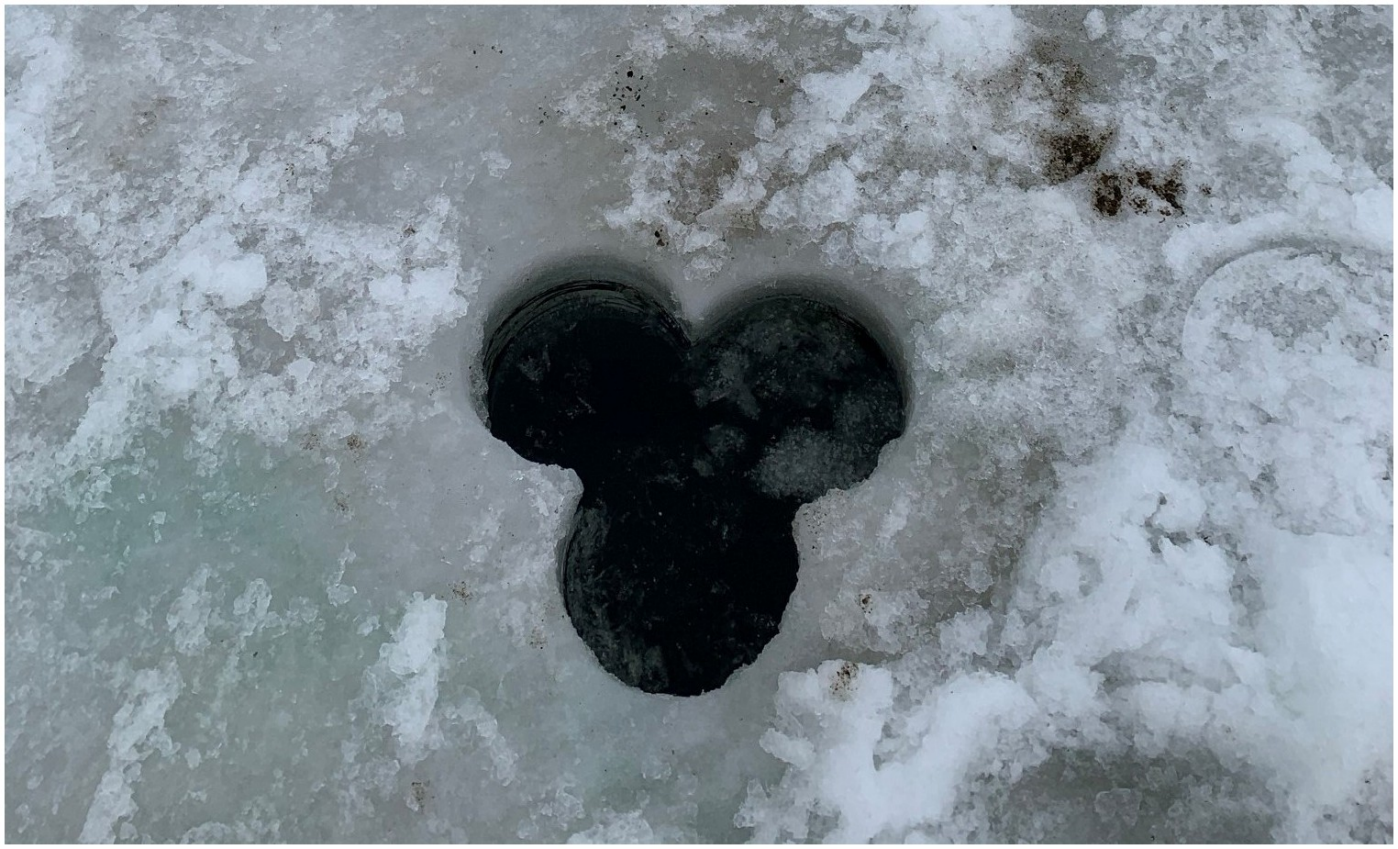
Holes for through-ice sampling. Image of the hole pattern drilled with ice auger to create large enough holes to accommodate D-net and scooping motions used in sediment sample collection.

When ice was present on the lake we used a gas-powered ice auger equipped with an 8-inch (20.32 cm) diameter blade to drill three connected holes in a triangular shape at each sample site to fit sampling equipment through (Fig 2). During open water sampling we accessed sites by boat or, in shallower, near-shore sites, by wading in the water to the sample site. At each sample site we used a D-net to scoop sediment, similar to a shovel, from the site into a large bucket (approx. 19 L). We then filtered the collected sediment through a 4.0 mm metal mesh sieve and removed any *C*. *fluminea* or empty *C*. *fluminea* shells captured in the sieve. For each individual or empty shell we recorded whether the specimen appeared to be living or dead and used a digital caliper to measure the shell length. Individuals were considered to be alive if they closed their shells during handling or if shells could not be opened with pressure was placed where valves meet at posterior and anterior ends. Shell fragments were not counted as we were not able to determine size of the full shell from fragments or how many individuals were represented by multiple fragments present in a single sediment sample. Individuals were returned to the sample site after measurements were recorded, with the exception of individuals recovered during our final sampling trip (September 23, 2021) which were removed from the system.

In addition to the individual counts and shell measurements, we recorded the GPS coordinates, water depth, and ice thickness (for through-ice sampling only) at each site. During winter sampling months we recorded water temperature, pH, dissolved oxygen, and conductivity at each sample site using a multiparameter water quality meter (YSI model 6920 V2, YSI Incorporated, 1700/1725 Brannum Lane, Yellow Springs, OH 45387) that was calibrated within one week of each site visit. Water quality data was only recorded at one location during open water sampling as there was not substantial variation in results across sites after ice-off and wind-driven boat drift made it difficult to ensure readings were taken from the actual sample site. All water quality readings were taken from the sediment-water interface to ensure recorded conditions were as close as possible to those experienced by the clams.

We created histograms using jamovi v1.6.23 [46] to determine the size classes present in Briggs Lake from both the winter sampling months and the end of the following summer. While combining multiple months of data from winter sampling was deemed appropriate as appreciable shell growth is not expected during the cold months [38, 47], we created the histogram for the end of summer analysis from a single sampling month as lumped data from the summer sampling would likely obscure the age classes due to ongoing shell growth during that time. We collected the end of summer data in September 2021 by adding an additional 33 haphazardly selected sample sites (total sites = 43) to our regular monthly monitoring to get a sample size large enough to create histograms of the clam size range.

## Results

**Fig 3:**
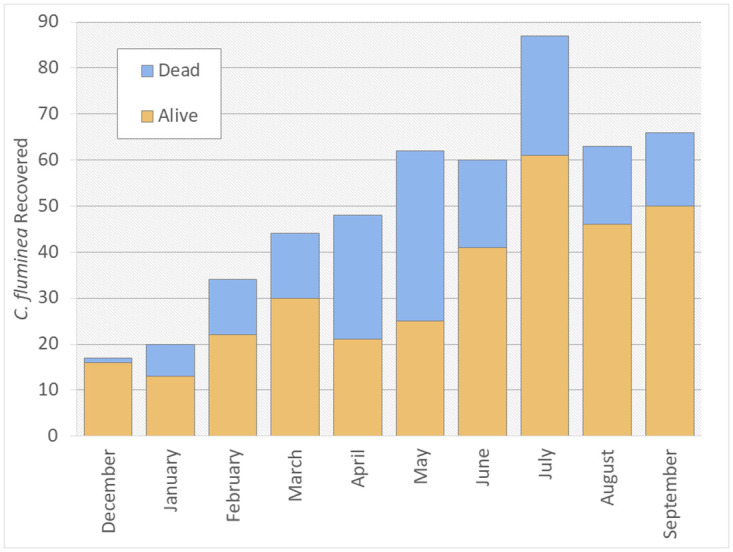
Monthly *C*. *fluminea* counts. Numbers of live (bottom, orange) and dead (top, blue) *C*. *fluminea* recovered during each sampling month.

We collected a total of 501 individuals (325 live, 176 dead) over the course of our regular monthly sampling and collected an additional 322 clams (252 live, 70 dead) as part of the additional September sampling for size class analysis. The percent of recovered *C*. *fluminea* that were alive at the time of sampling during regular monthly sampling ranged from 40.3% to 94.1% with a peak percent of dead *C*. *fluminea* (59.7%) recovered occurring in May 2021 (Fig 3).

**Fig 4:**
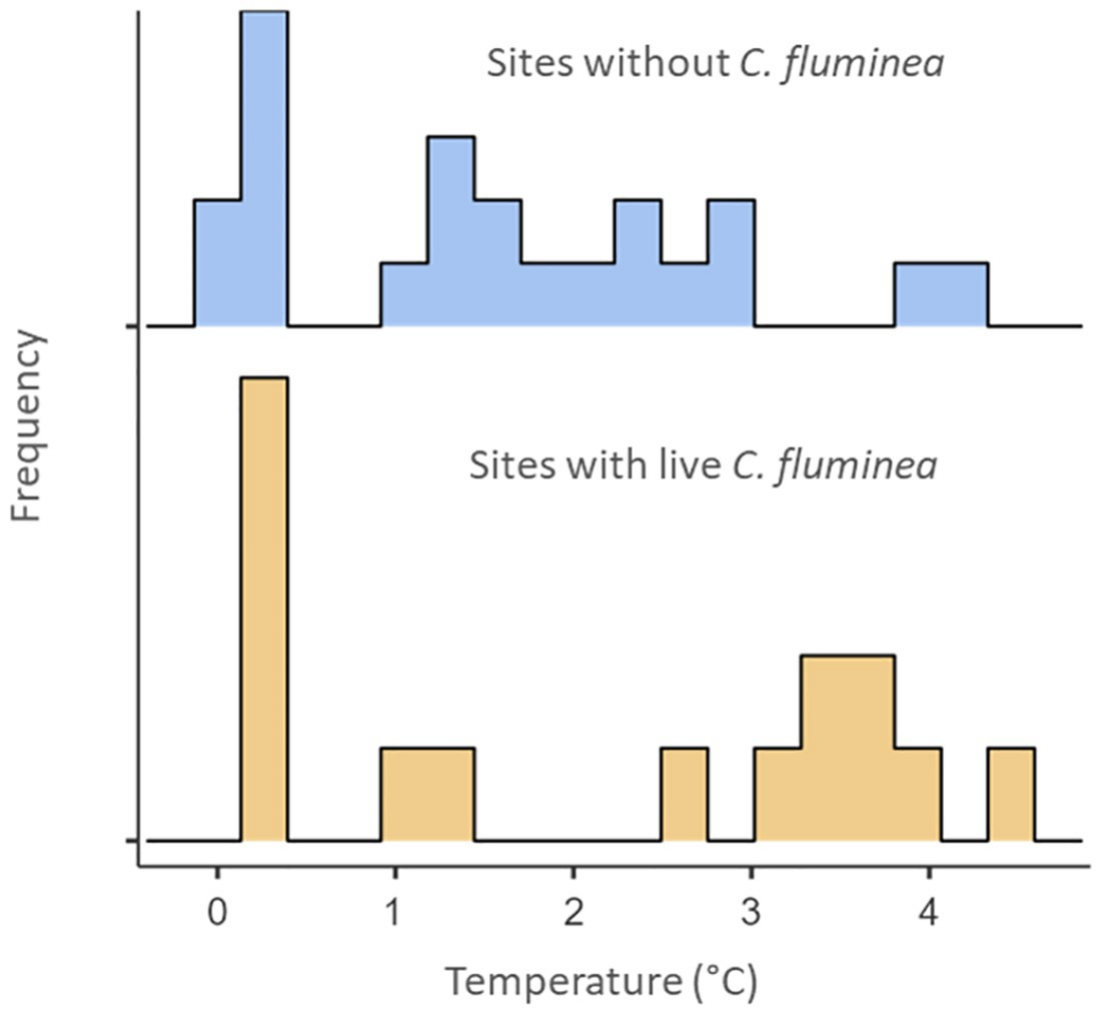
Winter temperature histogram. Histogram showing the frequency of individual monthly sample site temperatures (n = 37) during winter sampling activities at sites with (bottom, n = 15) and without (top, n = 22) live C. fluminea.

During the winter sampling months, water temperatures ranged from 0.3 °C to 4.5 °C, with an average winter temperature of 1.8 °C. Average temperatures in February and March were 1.9 °C and 0.3 °C, respectively, indicating multiple months with average temperatures below 2.0 °C conditions. Live clams were recorded at temperatures as low as 0.3 °C (Fig 4). March was the coldest month with a mean water temperature of 0.34 °C (*SD* = 0.02). This was likely due to the rapid ice melt occurring at the time with notable large cracks in the study area and a gap at the shoreline that would have allowed the fresh ice melt to mix in the water column. Mean water temperature from April-September was 20.0 °C (*SD* = 6.92, range 7.1 °C– 25.2 °C). Other environmental factors monitored during the study, including pH (range 7.5–8.8), conductivity (range 0.260 mS/cm—0.609 mS/cm), dissolved oxygen (range 2.2 mg/L– 12.7 mg/L), were within the permissive range for long-term survival of *C*. *fluminea* [48] (Table 1).

**Table 1:** Monthly mean (*SD*) environmental data.

Sample Month	Live/Dead *C*.*fluminea* Collected	Temperature (°C)	pH	Conductivity (mS/sec)	Dissolved Oxygen (mg/L)	Water Depth (m)	Ice Thickness (cm)
December	16/1	2.79 (0.92)	7.74 (0.73)	-^1^	11.9 (0.53)	0.74 (0.09)	19.5 (1.94)
January	13/7	2.29 (1.46)	8.26 (0.48)	0.473 (0.057)	9.18 (2.03)	0.75 (0.20)	25.2 (3.28)
February	22/12	1.91 (1.23)	7.59 (0.07)	0.555 (0.041)	3.65 (1.41)	0.80 (0.13)	49.0 (3.18)
March	30/14	0.34 (0.02)	7.87 (0.11)	0.261 (0.001)	11.2 (0.43)	0.84 (0.13)	37.6 (4.07)
April*	21/27	7.08	8.11	0.391	10.6	0.99 (0.24)	-
May*	25/37	20.1	8.23	0.360	9.89	0.77 (0.22)	-
June*	41/19	25.2	8.68	0.304	9.22	0.73 (0.19)	-
July*	61/26	24.5	8.54	0.302	9.42	0.65 (0.07)	-
August*	46/17	24.8	8.80	0.289	9.69	0.72 (0.10)	-
September*	50/16	18.5	8.35	0.309	8.08	0.79 (0.34)	-

^1^Conductivity probe malfunction resulted in no conductivity data collected in December.

*Temperature, pH, conductivity, and dissolved oxygen were recorded as single points from April–September.

**Fig 5:**
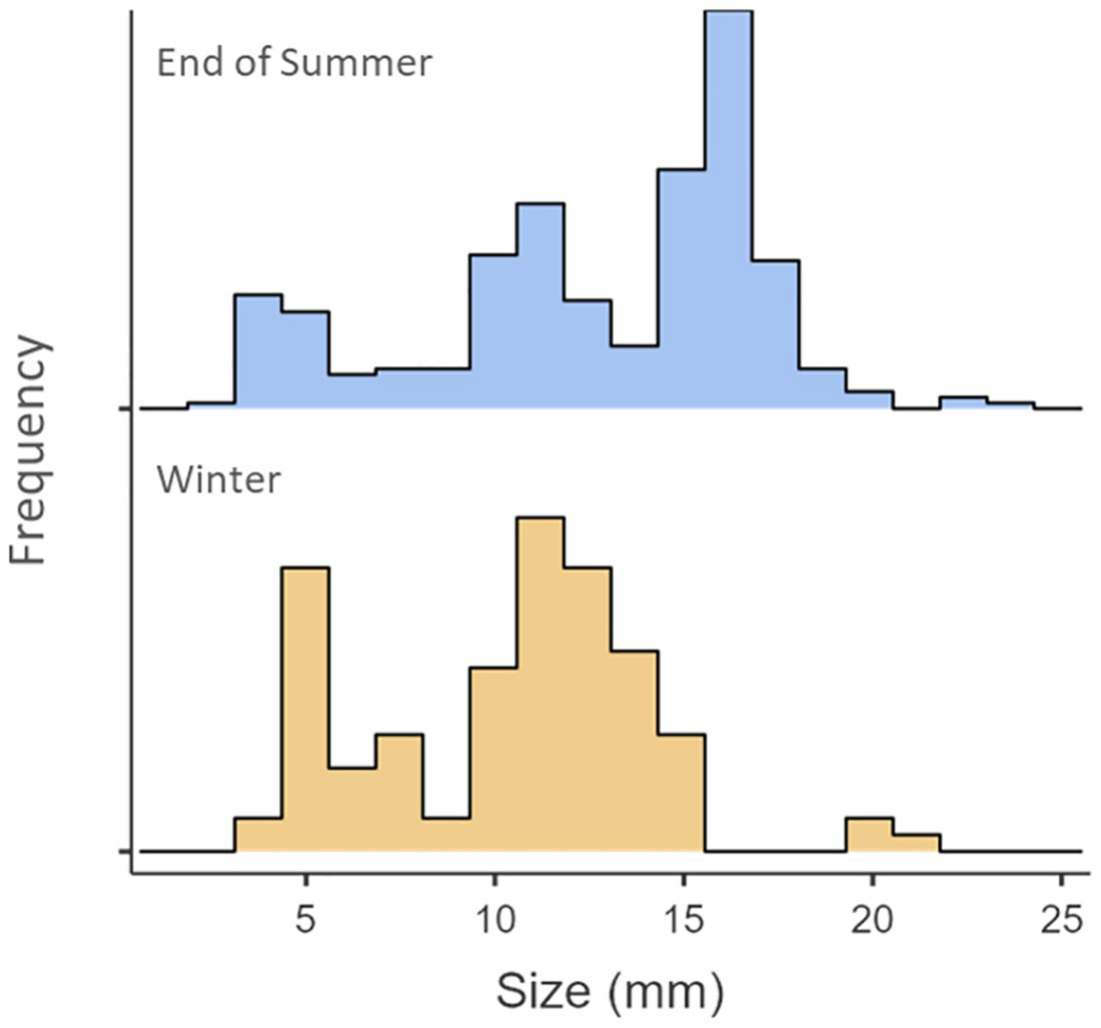
*C*. *fluminea* shell sizes. Histograms of live C. fluminea shell size recovered during the winter 2020–2021 sampling (bottom, n = 103) and end of summer 2021 sampling (top, n = 302).

Analysis of shell sizes revealed distinct size classes during the winter and summer sampling periods (Fig 5). The shell size histogram for live clams collected during winter sampling (December 2020 –March 2021) resulted in a bimodal distribution while the histogram for live clams collected at the end of the following summer (September 2021) resulted in a trimodal distribution (Fig 4). We identified natural breaks in the histograms to identify winter size classes with mean shell sizes of 5.75 mm (*SD* = 1.32 mm) and 12.1 mm (*SD* = 1.53 mm) with 3 larger individuals (19.83 mm, 19.94 mm, and 21.45 mm) which are likely surviving individuals from an older cohort. The end of summer sampling size classes were found with means of 4.46 mm (*SD* = 1.02 mm), 10.9 mm (*SD* = 1.52 mm), and 16.2 mm (*SD* = 1.16 mm) with 3 larger individuals likely from an older cohort (21.82 mm, 23.01 mm, and 23.36 mm).

## Discussion and conclusions

Understanding the climate tolerances of invasive species is of vital importance for predicting future distributions and accurate risk analyses. Our study shows that *C*. *fluminea* in Briggs Lake are surviving at temperatures below the previously established minimum temperature threshold of 2°C, at times near 0°C and are apparently reproducing following exposure to those temperatures. While we recorded live *C*. *fluminea* at temperatures below 2°C we often also recorded live clams at temperatures above 2°C throughout the winter months. Because we did not resample the same individuals or the same locations from month to month we are not able to determine how long an individual clam may have experienced these temperatures. This coupled with the variability across sample sites on a given day suggests that future in-situ studies would benefit from continuous data loggers to better understand the amount of time individual clams are exposed to temperatures below 2°C. Water temperatures at the water-sediment interface varied across the study area within single sample days and we recorded many sample sites with temperatures above 2°C throughout the winter, even with significant ice cover. Other studies have previously documented survival of *C*. *fluminea* in areas with extended ice cover which have led to suggestions that this species can likely survive in colder temperatures than previously thought [33, 34]. These observations help confirm that extended ice cover alone is not sufficient to describe the actual conditions and that direct water temperatures should be recorded at the water-sediment interface of sample sites to get a more accurate picture of the conditions benthic organisms are experiencing.

While the total number of clams recovered steadily increased each month during most of the study period, this is likely a result of our non-random sampling method rather than an indication of population growth during the study period. Our study did indicate relatively high clam survival throughout the study period, with live clams making up 40% to 94% of the recovered individuals each month. This stands in contrast to observations by Werner and Rothhaupt [39] where only 0.1% of *C*. *fluminea* in mesocosms that experienced ice cover for a shorter period of time than in our study and approximately 1% of clams at a lake field site survived into late spring or early summer. Similarly, Castañeda et al. [31] documented the complete extirpation of a well-established *C*. *fluminea* population in the St. Lawrence River following the permanent shut-down of the upstream power plant that had created the artificial thermal refuge the population survived in. We documented the lowest proportion of live recovered clams in the months of April and May. This could potentially be explained by observations by Cvetanovska et al. [41] which documented a lag period from exposure to cold temperatures and clam mortality such that survivorship remained high immediately following 8 weeks of exposure to cold temperatures but a significant drop in survivorship was observed after 8 weeks in recovery during lab trials.

Our winter size class analysis indicates there were already at least two size classes present in Briggs Lake during the 2020–2021 winter season. Existing literature provides a range of reproductive cycles in a year, though most suggest two peaks per year [19, 48–51]. This indicates *C*. *fluminea* has likely been present in Briggs Lake for at least 1 year prior to discovery in August 2020. The two larger size classes in the end of summer sampling are likely the same cohorts that we identified from winter sampling and represent the size increases of those cohorts throughout the summer months. The smallest size class in the end of summer sampling is likely recruitment from a reproductive event during the 2021 warm water season. Similar patterns in size classes were recorded in a South American population of *C*. *fluminea* by Ituarte [47] which documented a monthly shift of existing cohorts as they grew over time and recruitment of new cohorts appearing on size histogram plots. Our observed rate of single season shell growth is comparable to those observed by Weitere et al. [52]. Despite the 4 mm size of our sieves we were still able to capture some individuals smaller than the sieve size, providing further evidence of reproduction during the 2021 season through the recovery of individuals as small as 2.2 mm in September 2021.

The Briggs Lake population appears to be the northernmost documented population of *C*. *fluminea* in North America outside of a thermal refuge with the exception of the populations in Washington, USA; British Columbia, Canada; and at Lake Elmo, Montana, USA [22, 25]. Peel et al.’s [53] updated Köppen-Geiger climate type map highlights a key difference in these northern latitude exceptions, which are located in areas generally dominated by temperate climate types while all of Minnesota is covered by cold climate types. Minnesota was not found to be a suitable climate match for *C*. *fluminea* under current climate conditions in modeling done by McDowell et al. [23] and Gama et al. [24]. These two studies use different modeling approaches, but both rely on known distribution data to train the models. The McDowell et al. model determined some locations from previous studies documenting live *C*. *fluminea* in colder climates such as Connecticut [38], New Hampshire [36], New York [35] and southern Michigan [54] as suitable habitat under present conditions (for some even under more conservative modeling). This suggests that modeling can overcome shifts in climate tolerance for species or provide suggestions for suitable habitat that current knowledge about thermal limits for a species may otherwise ignore. However the presence and apparent reproduction of *C*. *fluminea* documented in our study in a region the McDowell et al. model doesn’t consider suitable habitat under current (as of 2012) climate or 2 of 3 projected year 2080 climate conditions demonstrates a need to refine these models to better understand the potential risk this species poses both under current climate conditions and future climate scenarios. These models can be important tools to help inform regulatory decision-making on if and how to manage non-native species. For example Minnesota state statute requires the consideration of “the likelihood that the species would naturalize in the state if it were introduced” as part of Minnesota Department of Natural Resource’s non-native species classification process (Minnesota Statutes 2020 84D.04).

Further evidence that *C*. *fluminea* can survive and reproduce in central Minnesota and likely tolerate temperatures below 2°C surfaced with the discovery of a live specimen at a second inland lake in central Minnesota approximately 23 km from our study location (Big Lake, Sherburne County, Minnesota, 45.334076°N, 93.756191°W) during Starry Trek in August 2021. We visited this location in September 2021 and uncovered 15 live clams between 7.13 mm and 16.97 mm in size. The clams were mostly concentrated around the public boat launch, however two of the live individuals were found in the adjacent swimming area up to approximately 150 m from the boat launch. We also observed many (estimated over 100) empty shells in this area. While Minnesota Department of Natural Resources staff recorded dead (empty) shells in Big Lake in 2019 and 2020 they never recovered any living specimens [55]. Big Lake is notably different from Briggs Lake in that it is mesotrophic, although the two lakes do share a primarily sandy substrate in the shallow waters. A likely cause for the extreme differences in number of live versus dead clams is that the entire area surrounding the swimming beach and boat launch is regularly treated with copper sulfate targeting snails to limit swimmer’s itch [56].

We speculate that the overwinter survival and apparent reproduction in these two central Minnesota lakes are evidence of the evolution of increased cold tolerance at the northern invasion front of *C*. *fluminea*. Laboratory trials by Cvetanovska, et al. [41] indicated that *C*. *fluminea* from colder climates were more likely to tolerate exposure to temperatures near freezing temperatures with acclimation than individuals collected from warmer climates. Evolution of cold tolerance at invasion fronts have been observed in other invasive species including an invasive house gecko (*Hemidactylus frenatus*; [57]), cane toads (*Rhinella marina*; [58]), elongate hemlock scale (*Fiorinia externa*; [59]), and mosquitofish (*Gambusia affinis*; [60]) and lab experiments have demonstrated the ability of another invasive mollusc, the apple snail (*Pomacea canaliculata*) to develop improved cold tolerance with acclimation [61, 62]. *C*. *fluminea*’s unique reproductive strategy may also provide advantages in its invasion successes and the establishment of populations with higher tolerance of environmental conditions, like temperature [16]. While the Briggs Lake population was the first record of live *C*. *fluminea* in an inland Minnesota lake with no thermal refuge, *C*. *fluminea* has been present in Minnesota river systems since the late 1970s, [63] typically associated with thermal plumes from power plants or wastewater treatment plant outfalls [22]. One of the reported populations in the Mississippi River noted by Benson and Williams [22] can be found near the Monticello Nuclear Generating Plant within 20 km of both the Briggs Lake and Big Lake populations, though it is unknown what the source population for these lakes is. The possibility of these kinds of thermal plumes creating environments that promote adaptation of cold tolerance and the risks that may pose to nearby unheated water bodies at these invasion fronts warrants further research attention.

Finally, we would like to note the importance of an engaged public in the early detection of aquatic invasive species [64]. The initial discovery of *C*. *fluminea* in Briggs Lake was made thanks to the sharp eyes of a young volunteer participating in a volunteer aquatic invasive species monitoring event (Starry Trek) in August 2020 and the subsequent discovery of a live individual in Big Lake was made by another volunteer participating in the same event in August of 2021. This event has led to volunteers making first reports of previously unknown populations of starry stonewort (*Nitellopsis obtusa*), zebra mussels (*Dreissena polymorpha*), Eurasian watermilfoil (*Myriophyllum spicatum*), and other invasive species in Minnesota, some of which have resulted in rapid response management efforts [65–67], and ongoing and new research efforts. Engaged members of the community have made other important invasive species discoveries in Minnesota and beyond [44, 64, 68–70]. We continue to encourage public outreach campaigns and organized volunteer programs that can discover new invasive species populations to inform future management and research. In addition, we hope future research will explore improved modeling to better understand the potential range of *C*. *fluminea*, better understanding of the potential for establishment and impacts of *C*. *fluminea* in cold climate zones, and the potential of thermal plumes to serve as habitat for bridgehead populations where adaptation can occur to allow for further introductions [71] in cold climate zones.
